# The Effects of Orthodontic Brackets on the Time and Accuracy of Digital Impression Taking

**DOI:** 10.3390/ijerph18105282

**Published:** 2021-05-16

**Authors:** Hyojin Heo, Minji Kim

**Affiliations:** 1 Department of Preventive Dentistry, Kyungpook National University School of Dentistry, Daegu 41940, Korea; hyojin7651@gmail.com; 2 Department of Orthodontics, School of Medicine, Ewha Womans University, Seoul 07985, Korea

**Keywords:** digital impression, measuring time, orthodontic brackets, scanner accuracy

## Abstract

*Background:* The aim of the study was to study how the presence or the type of the orthodontic brackets influence the time measurement and accuracy of impressions using a digital oral scanner. *Methods:* The same models were divided into the control group (the model without a bracket), MB group (the model with a metal bracket), and CB group (the model with a monocrystalline bracket). Subsequently, scanning was conducted five times for each model using the Trios Pod 2^®^. Simultaneously, the duration for taking the digital impression was measured. The degree of accuracy was compared among the three groups. *Results:* As compared with the control group, scanning took 53.3 s longer in the MB group and 194.23 s longer in the CB group. In the canine and the first molar, the mean values of errors were compared between the left and right sides; in both the canine and the first molar, errors between the control group and the CB group were the greatest. *Conclusions:* Following a comparison of the duration and accuracy of the impressions between the three groups, our results suggest that its degree was the highest in the CB group where a monocrystalline bracket was attached.

## 1. Introduction

In the field of dental practice, a tooth model is used in the process of reconstructing the number and size of teeth, tooth anomalies, and adjacent tissues [1]. Thus, it helps to establish a diagnosis and formulate a treatment plan. In particular, the tooth model is key to an accurate diagnosis, determining treatment modalities, course, and prognosis prior to the initiation of orthodontic treatment [2].

Conventionally, a tooth model is prepared after taking an impression of the oral cavity using impression materials. Therefore, the time required to take impressions varies, depending on the types of impression material used [3]. There are disadvantages associated with the use of impression materials, such as patient discomfort, including gag reflex; the possibility of deformity due to impression materials; and difficulty in storing a plaster cast [4,5]. With recent technical advancements in oral scanning, digital impression taking methods have been frequently used in the clinical setting. Unlike the conventional tooth models, digital models allow more accurate measurements and faster analysis. It can therefore be applied as a diagnostic tool as well. Furthermore, it allows for the ease of storage [6,7,8]. However, to prepare a digital model in a problem-free manner, certain time needs to be devoted to learn the methods for using an oral scanner. Moreover, proficiency and clinical experience is required on how to acquire tooth impressions or on the treatment of saliva and gingival crevicular fluid before taking impressions of the subgingival margin, and the learning curve should be considered in terms of accuracy [9,10].

With the technical advancement in a variety of oral scanners, many studies have been conducted to analyze the accuracy of digital images. Previous studies have compared the tooth width measurement on a digital model, obtained using an oral scanner, in a clinical setting and the actual width measured on a plaster cast and reported no significant difference between the two measurements [3,11,12]. These studies also compared the duration of taking impressions using an irreversible hydrocolloid impression material and that acquired digitally. In a clinical setting, the duration of impression taking was shorter in cases of the alginate. However, considering the time taken to prepare the model, the duration of the oral scanner was shorter; this duration indicates the efficiency of the procedure. Yuzbasioglu et al. [13] measured the duration of impression taking using a rubber-base impression material and an oral scanner, thus comparing the time efficiency between the two materials. In addition, Lee et al. [14] also measured the duration of impression taking using a rubber-base impression material and an oral scanner for taking the impression for implant abutment. Both studies have shown that the duration of impression taking using an oral scanner was shorter. Furthermore, comparing the scanning results between the metal, resin, and ceramic models, a difference in the degree of light reflection was observed depending on the type of material and the angle of scanning [15].

In the field of orthodontics, the tooth model is used as a diagnostic aid before initiating orthodontic treatment. Furthermore, the tooth model can also be used to prepare orthodontic appliances, transfer jig for indirect adhesion, and custom prescription brackets [16]; evaluate occlusal stability [17,18]; and to facilitate orthopedic-orthodontic treatment planning [19]. Therefore, impression taking is an inevitable procedure for the preparation of tooth model from the period before initiation of the orthodontic treatment to until its completion.

In patients with bonded orthodontic brackets, an impression taking session using an impression material is a complex and uncomfortable procedure; it is uncomfortable for both dentists and patients [13,20]. With the increased use of digital oral scanners, various orthodontic studies have been conducted to examine the accuracy and reproducibility of digital impression taking for the dental arch [3,11,12,13,14,15,16,17,18,21,22]. In previous in vitro studies, the accuracy of impressions taken using four digital oral scanners of dental arches with calibration brackets were compared, and all oral scanners showed variable accuracy in horizontal measurements [23]. An in vivo study compared seven digital oral scanner and digital impression systems, all within the range allowed by the digital workflow, and demonstrated differences in precision [24]. The digital intraoral impression system continues to develop rapidly. Recent studies have evaluated the effect of the presence of orthodontic brackets [25,26,27,28,29]. However, there is still a lack of digital impression research on the effect of the type of orthodontic bracket.

This study examined the effects of the presence and types of the orthodontic brackets on the duration and accuracy of impression taking during the application of a digital oral scanner. The null hypothesis stated that the existence and type of orthodontic brackets would have no significant difference on the time and accuracy of oral scanner measurement.

## 2. Materials and Methods

### 2.1. Experimental Models

**Figure 1:**
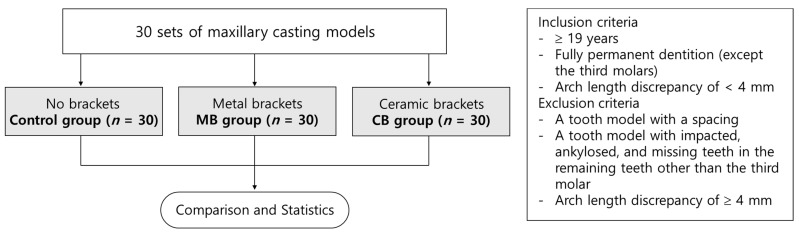
Flow chart of study protocol.

The study protocol is outlined in the flow chart shown in Figure 1. The sample comprised 30 sets of maxillary models obtained from 30 different patients, which were duplicated three times and used for each group. The sample size was determined by adding values from a previous study using G*Power (version 3.1, Universität Kiel, Kiel, Germany) [30,31]. The inclusion criteria were as follows: (1) a minimum age of 19 years; (2) fully permanent dentition (except the third molars); and (3) a tooth model with an arch length discrepancy of <4 mm. The exclusion criteria were as follows: (1) a tooth model with a spacing; (2) a tooth model with impacted, ankylosed, and missing teeth in the remaining teeth other than the third molar; and (3) a tooth model with an arch length discrepancy of ≥4 mm.

All the plaster cast models had good quality and were accurately and uniformly trimmed with their bases parallel to the occlusal surface [32].

### 2.2. Attachment of the Bracket to the Model

**Figure 2:**
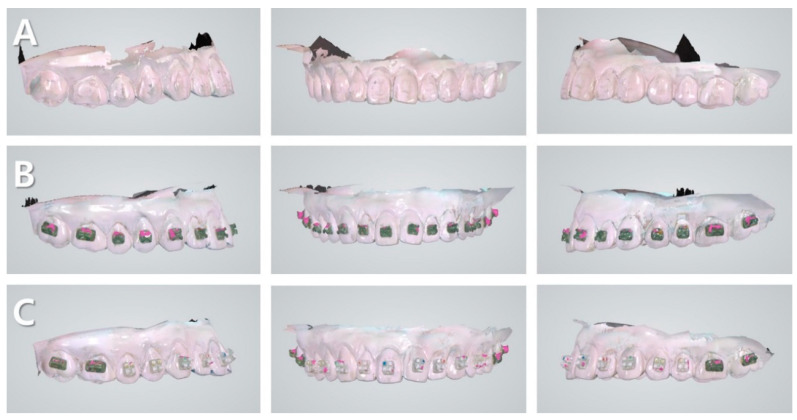
Scan image of the experimental on Trios Pod 2^®^. (**A**) Control group (the model without a bracket), (**B**) MB group (the model with a metal bracket), and (**C**) CB group (the model with a monocrystalline bracket).

The 30 identical maxillary models were categorized into the following three groups: the group where no brackets were attached, that where metal brackets were attached, and that where ceramic brackets were attached. The metal brackets used were the Mini Uni Twin Bracket™ (3M Unitek, Monrovia, CA, USA). The ceramic brackets used were monocrystalline brackets (Bijou Bracket™, World Bio Tech, Seoul, Korea). All the brackets were of the MBT type (018 slot). Metal and ceramic brackets were attached to the same location using Transbond^TM^ XT (3M Unitek, Monrovia, CA, USA) without etching, primer, and bonding processes [33,34]. The group where no brackets were attached, that where metal brackets were attached, and that where ceramic brackets were attached were termed as the “control group,” “MB group,” and “CB group,” respectively (Figure 2).

### 2.3. Study Methods

#### 2.3.1. The Measurement of the Scanning Duration and Impression Taking Depending on the Presence and Types of Orthodontic Brackets

Scanning was performed by a single investigator who had an experience of using the second generation of Trios Pod 2^®^ (3Shape, Copenhagen, Denmark) [35] for more than 20 times a week, over more than 3 months after sufficient training. In consideration of measurement errors, measurements were repeated five times for each model, averaging the other three figures except the maximum and minimum values [25]. In addition, to rule out the effects of investigator fatigue, the daily frequency of digital impression taking was limited to 10 [9,36]. The models of the control, MB, and CB groups were scanned five times each at 10 min intervals.

Scanning was performed from the left second molar to the right one, partially involving the lingual and occlusal surface. After scanning the occlusal surface extending from the right second molar to the left one the buccal surface was scanned extending from the left second molar to the right one. Thus, the primary scanning was concluded. This was followed by the additional scanning for the areas where no scans were obtained. When the tooth and its adjacent tissue had less than six holes with a diameter of <2 mm on Trios Pod 2^®^ scans, we determined that scanning procedure was completed. The corresponding time points were considered at the end of the scanning. Impression taking with an oral scanner was performed solely for the maxillary model [37], and the duration of the primary and additional scanning procedures was included.

In each group, the mean and maximal duration of the impression taking was compared for the duration of scanning for 30 models, five times at a unit of seconds.

#### 2.3.2. Comparison of the Accuracy Depending on the Presence and Type of the Orthodontic Brackets Overlapping of the Digital Models

All data were converted to the Standard Tessellation Language (.stl) format using the OrthoAnalyzer^TM^ software (Version 2013, 3Shape, Copenhagen, Denmark). Of the digital models that were scanned five times, the last scanned file was used for overlap. The initial registration was completed by selecting three points on the corresponding images obtained in each group. The three points considered during initial registration were the mesiobuccal cusp tips of the right and left second molars, and the mesiolabioincisal point angle of the right central incisor [38]. In addition, the surface except for the orthodontic brackets was designated as a reference, and the automatic “regional register” functions of the Rapidform^TM^ (Version 2004, INUS Technology Inc., Seoul, Korea) were used.

Overlapping of the STL files in the three groups was repeated three times, and the smallest deviation value was used in statistical analysis. Absolute values were used as measurements in this study. Therefore, a total of 30 overlapped images were obtained.

#### 2.3.3. Deviations among the Three Groups

**Figure 3:**
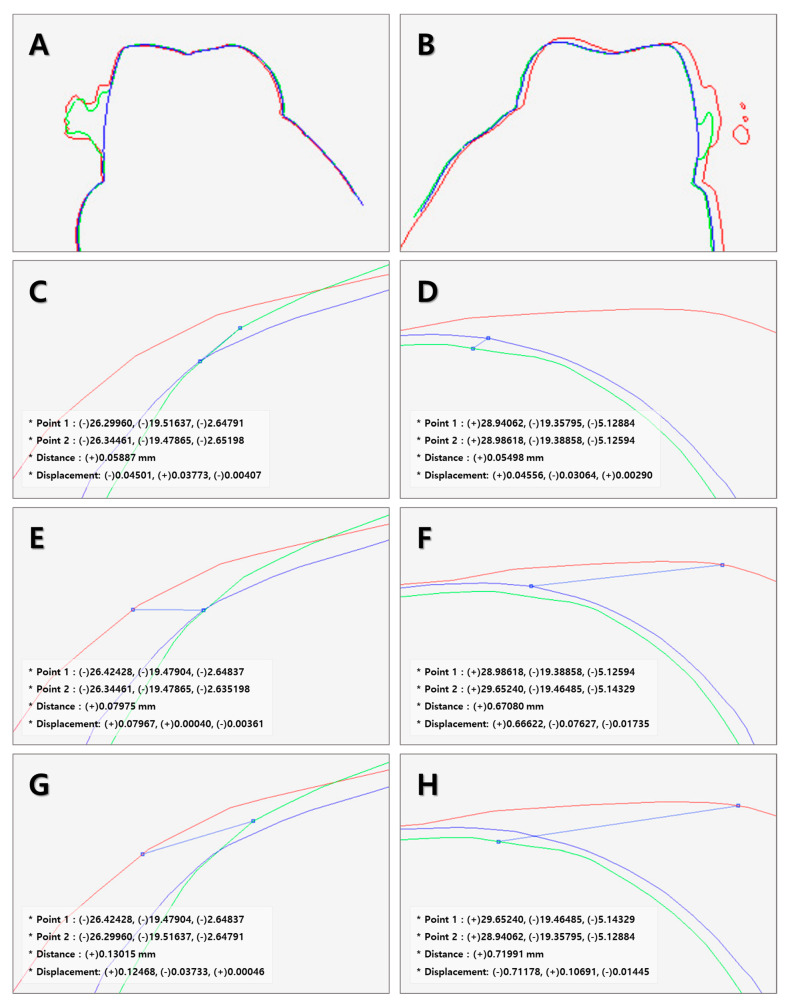
The deviation of the measured model with Rapidform 2004 ^TM^. Blue line = control group, green line = MB group, red line = CB group. (**A**,**B**) 2D cross-section of first molar mesiobuccal cusp, (**C**,**D**) distance between the control group and MB group on first molar mesiobuccal cusp, (**E**,**F**) distance between the control group and CB group on first molar mesiobuccal cusp, and (**G**,**H**) distance between MB group and CB group on first molar mesiobuccal cusp.

Using the “shell/shell deviation” functions of the Rapidform^TM^ (Version 2004, INUS Technology Inc., Seoul, Korea), differences between the three groups were analyzed on four cross-sections. The four cross-sections were perpendicular to the occlusal plane, coinciding with the tip of the left and right canines and the mesiobuccal cusp of the first molar. The tip of the canine and the mesiobuccal cusp of the first molar served as landmarks [25], based on which the distance between the landmarks was defined as the difference in cross-sections of the overlapped images. On cross-sections, the distances between the landmarks between the control and MB groups, control and CB groups, and MB and CB groups were measured. At the left and right landmarks, errors and their mean values were calculated (Figure 3).

### 2.4. Statistical Analysis

The statistical analysis was performed using IBM SPSS Statistics version 18.0 for Windows (IBM corp., Armonk, New York, NY, USA), and the significance level was set to 0.05. To analyze the statistically significance differences in the duration of impression taking and the accuracy between the control, MB, and CB groups, one-way analysis of variance (ANOVA) was performed. If there was a difference in the mean analyzed by one-way ANOVA, a post hoc test was performed with the Duncan test to confirm the difference between the groups. Intra-rater reliability analysis was performed using intraclass correlation coefficients (ICC). ICC range from 0.0 to 1.0 (0.00–0.49, poor; 0.50–0.74, moderate; 0.75–0.90 good; and ≥0.90, excellent agreement [39]).

## 3. Results

### 3.1. Comparison of the Duration of Scanning and Impression Taking According to the Presence and Types of Orthodontic Bracket

A total of 30 models in the control, MB, and CB groups were scanned five times. This was followed by a comparison of the mean and maximum values of the duration of the impression taking (Table 1). This showed a significant difference between the three groups (*p* < 0.001).

The mean duration for taking the impression was 243.10 s in the CB group, 102.17 s in the MB group, and 48.87 s in the control group. This indicates that it was the longest in the CB group where monocrystalline brackets were attached and the shortest in the control group where no brackets were attached. Compared with the control group, in the MB group, the duration of the impression taking was longer by 53.3 s. Compared with the control group, in the CB group, the duration of impression was longer by 194.23 s. The maximal duration of impression taking was 267.13 s in the CB group, 115.03 s in the MB group, and 65.23 s in the control group. This indicates that it was the longest in the CB group where monocrystalline brackets were attached and the shortest in the control group where no bracket was attached. Thus, the null hypothesis had to be rejected.

### 3.2. Comparison of the Accuracy According to the Presence and Types of Orthodontic Brackets

The ICC values for the canine and first molar measurement are presented in Table 2. The ICC value of the right canine was 0.99 and that of the left canine was 0.97. It was 0.94 for the right first molar and 0.93 for the left first molar, indicating that the measurement of canine and first molar showed excellent reliability.

**Figure 4:**
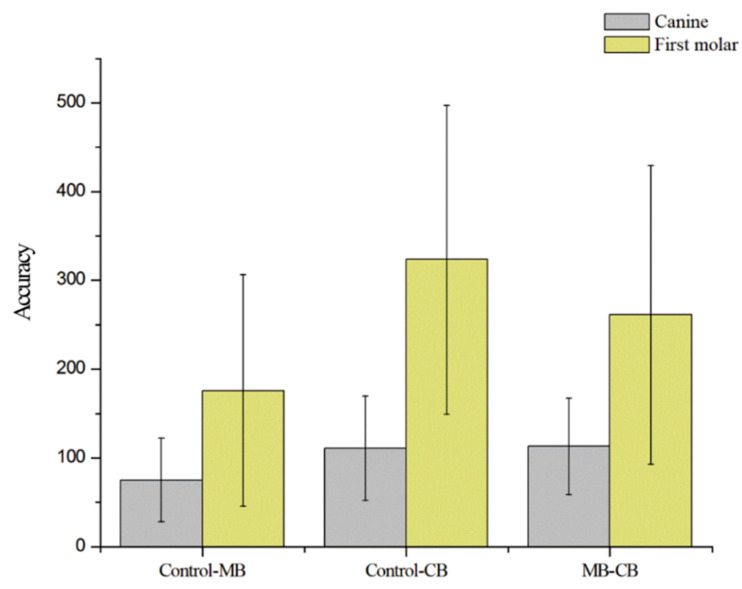
Average deviations on scanned images according to buccal bracket. Control = control group (the model without a bracket); MB = MB group (the model with a metal bracket); CB = CB group (the model with a monocrystalline bracket); Control-MB = distance between control group and MB group on canine tip and first molar mesiobuccal cusp; Control-CB = distance between control group and CB group on canine tip and first molar mesiobuccal cusp; MB-CB = distance between MB group and CB group on canine tip and first molar mesiobuccal cusp.

The STL files of three groups of models were overlapped to determine the deviation between the presence and type of orthodontic bracket, which was measured in micrometer. The digital models were overlapped and the mean values of deviation at the cusp tips of the right and left canines, and at between the mesiobuccal cusp tips of the right and left first molars were compared (Figure 4 and Table 3); no significant differences were observed among the right and left canines and the right first molar. The first molar on the left showed a significant difference of 176.34 μm in deviation between the control and MB groups, 385.99 μm between the control and CB groups, and 305.48 μm between the MB and CB groups (*p* < 0.05).

The values were found to be 74.90 μm between the control and MB groups, 110.90 μm between the control and CB groups, and 112.95 μm between the MB and CB groups. At the mesiobuccal cusps of the first molars, the mean values of deviation were 175.69 μm between the control and MB groups, attaining statistical significance, 323.32 μm between the control and CB groups and 261.14 μm between the MB and CB groups. According to the results of the Duncan test, there was a significant difference in the mean values of deviation between the control and MB groups. This is because displacement was the largest in the CB group.

## 4. Discussion

We examined the effects of the presence and type of orthodontic brackets on the duration and accuracy of digital impression taking using an oral scanner. In this study, the tooth models were categorized into three groups where no orthodontic brackets were attached, where metal brackets were attached, and where ceramic brackets were attached. Ceramic brackets can be generally classified into polycrystalline and monocrystalline brackets [1,40]. The degree of light transparency of ceramic brackets is higher but they are fragile due to the size of crystalline particles, ranging from large crystalline particles to ultrafine structural perspectives [40]. In this study, monocrystalline brackets with the highest degree of transparency were used.

This study used the second generation of Trios Pod 2^®^ as an oral scanner based on a confocal laser scanning method. Based on the methods for capturing moving pictures, the entire mouth is continually overlapped and then scanned in a single session. The mean duration of the impression taking was 48.87 s in the control group, 102.17 s in the MB group, and 243.10 s in the CB group. Variability in the duration of the digital scanning using an oral scanner was seen depending on the attachment and type of brackets. Particularly, in the CB group wherein the monocrystalline bracket was used, the duration of the impression taking was relatively longer. This is because the bracket has excellent transparency and the scanner did not perceive the light due to the light scattering, though it used a light source [15,41].

The scan was carried out in accordance with the procedures recommended by the user manual. The overlapping deviations of measurements on the models of the three groups, the left and right canines, and the right first molar were not significantly different, but there were significant differences of 209.65 μm in the left first molar (*p* < 0.05). Ender et al. [21,42] and Patzelt et al. [31] said that overlapping software of intraoral scanners could cause errors, especially in the posterior region due to horizontal expansion, and Anh et al. said that errors were observed in the posterior region, opposite the intraoral scanner starting point [43]. Likewise, this study also showed a significant deviation in posterior landmark measurements.

As the factors affecting the accuracy of the oral scanner images would be affected by the angular accuracy providing basic information for the acquisition of images and environmental factors such as the surface reflection rate, temperature, and humidity [15,35,44], there was a slight difference depending on the type of the material. However, it was shown that ceramic had a lower degree of accuracy than metal. To resolve the problem that an oral scanner cannot perceive the light due to the light reflection of the material, methods such as using a spray powder can be considered [45].

For the analysis of the three-dimensional data obtained using digital impression taking, overlapped images of the three groups were compared by specifying the difference in the distance as the error at the left and right canine tips and the mesiobuccal cusps of the left and right first molars on two-dimensional images. The distance between the canines and that between the posterior teeth, both of which are horizontal measurements that have been mainly used for a conventional type of analysis of orthodontic models, would vary depending on the location or angle of the digital model. It is also difficult to accurately assess three-dimensional models on two-dimensional images. It was therefore determined that minute errors could not be evaluated accurately [11]. At the tips of the left and right canines and the mesiobuccal cusp of the first molar, the mean values of errors were compared between the control and MB groups, control and CB groups, and MB and CB groups, which showed differences. In both the canines and first molars, the smallest difference was evident between the control and MB groups. This suggests that the displacement was the largest in the CB group where the monocrystalline bracket was attached.

According to the previous studies about the accuracy of oral scanners, the accuracy of the intraoral scanner was 20 μm when scanning was done in an extraoral setting and 50 μm in an intraoral setting. However, the results of their studies were not affected by the prosthesis or the bracket [16,46]. Zilberman et al. [47] reported that there were errors ranging from 70 to 330 μm, which could be applied to a clinical setting. Errors between the groups other than those between the control and CB groups in the first molar could be applied to a clinical setting. However, errors between the control and CB groups were 323.32 μm; these values could not be applied to a clinical setting for which a meticulous workup would be needed [16,46,47,48] However, the Trios Pod 2^®^ can directly confirm the images obtained during digital impression taking. Regardless of the error, it can be inferred that there will be no problem visually explaining to the patient on the chairside.

In this study, during the impression taking using an oral scanner, relative to the MB group in which metal brackets were attached, the duration of the impression taking was the longest in the CB group. Moreover, it was also shown that the degree of accuracy became lower. Based on these results, it can be inferred that transparent brackets, such as monocrystalline brackets, would have more detrimental effects on intraoral scanning and preparation of digital models, as compared with metal brackets with undercuts and a certain degree of light reflection.

In the present study, the tested data set were obtained from “.stl” files. Impression data obtained by oral scanners typically use “.stl” files, which have only black and white morphological information about the intraoral geometry. In comparison, “.obj” and “.ply” files have information such as color, texture, and transparency in addition to morphological information on shapes in the mouth, which helps distinguish teeth from surrounding soft tissues [49]. The scope of extraction of supported file types may vary depending on the oral scanner; so, this should be considered depending on the scope of use.

This study has several limitations. First, we did not use models of various cases. Second, the plaster cast model was used for taking digital impressions. Limitations in the oral cavity were not considered. In the oral cavity, it is difficult to access the distal surfaces of the posterior tooth. The degree of accuracy would be affected by factors such as saliva, blood, patients’ cooperation, and the orthodontists’ technical expertise [26,50]. Hence, further studies conducted in an actual oral cavity setting should be planned. Notwithstanding these limitations, this study was valid enough to provide information about the scope and limitations of the use of an oral scanner.

Based on these results, in cases in which a meticulous workup is needed, such as the preparation of the brackets and wires through a set-up of the virtual tooth model, the effects of the type of brackets during digital impression taking should be considered [51,52]. In addition, if it is possible to establish the methods for applying digital impression taking during orthodontic therapy, patients would be given treatments more conveniently and quickly. In doing so, the rationale of taking digital impressions and the approximate duration of impression taking can be explained to patients. Thus, a trustworthy relationship can also be established between doctors and patients.

## 5. Conclusions

This study explored how the presence and type of orthodontic brackets influenced the time and the accuracy of taking digital impressions. In particular, in cases of a transparent material, such as monocrystalline brackets, the degree of difference in measurements was relatively higher than that with metal brackets. Changes depending on the presence and type of brackets are located mainly by posterior teeth, which should be considered during the use of an oral scanner in a clinical setting.

## Figures and Tables

**Table 1:** Comparison of intraoral scanner recording time according to orthodontic brackets (unit: s).

Recoding Time	Control	MB	CB	*p*-Value *
Mean	48.87 ± 7.26 ^c^	102.17 ± 10.61 ^b^	243.10 ± 34.98 ^a^	<0.001
Max	65.23 ± 9.12 ^c^	115.03 ± 14.42 ^b^	267.13 ± 54.54 ^a^	<0.001

*n* = 30, * *p* < 0.05 by one-way ANOVA. Control = control group (the model without a bracket); MB = MB group (the model with a metal bracket); CB = CB group (the model with a monocrystalline bracket). ^abc^ same letters means there is significant difference between groups by one-way ANOVA.

**Table 2:** Results of intra-rater reliability analysis for measured canine and first molar using ICC.

Tooth			ICC (2,1)	95% CI		*p*-Value *
				Min	Max	
**Canine**	Right	Measurements	0.990	0.940	1.000	0.000
	Left	Measurements	0.965	0.818	0.999	0.000
**First molar**	Right	Measurements	0.936	0.732	0.998	0.000
	Left	Measurements	0.933	0.719	0.998	0.000

* *p* < 0.05 by ICC. Min = minimum; Max = maximum; CI = confidence interval.

**Table 3:** Average deviations of right and left reference points on scanned images according to buccal bracket (unit: μm).

Variable	Mean ± SD					
	#13	#23	Canine	#16	#26	First Molar
Control-MB	76.69 ± 65.13	73.11 ± 68.31	74.90 ± 47.05 ^b^	175.03 ± 195.52	176.34 ± 151.04 ^b^	175.69 ± 130.22 ^b^
Control-CB	104.29 ± 75.49	111.52 ± 95.35	110.90 ± 58.70 ^a^	260.05 ± 233.21	385.99 ± 255.99 ^a^	323.32 ± 173.90 ^a^
MB-CB	99.37 ± 74.61	126.53 ± 105.18	112.95 ± 54.41 ^a^	216.80 ± 190.24	305.48 ± 236.30 ^a^	261.14 ± 167.99 ^a^
*p*-value *	0.289	0.057	0.011 *	0.283	0.002 *	0.002 *

* *p* < 0.05 by one-way ANOVA. Control = control group (the model without a bracket); MB = MB group (the model with a metal bracket); CB = CB group (the model with a monocrystalline bracket); Control-MB = distance between control group and MB group on canine tip and first molar mesiobuccal cusp; Control-CB = distance between control group and CB group on canine tip and first molar mesiobuccal cusp; MB-CB = distance between MB group and CB group on canine tip and first molar mesiobuccal cusp; #13 = upper right canine; #23 = upper left canine; #16 = upper right first molar; #26 = upper left first molar. ^ab^ same letters means there is significant difference between groups by one-way ANOVA.

## Data Availability

Data are contained within the article.
